# Anasarca, Fever, Thrombocytopenia, Organomegaly, and Multiorgan Failure in a 24-Year-Old Pregnant Woman

**DOI:** 10.1155/2017/3871593

**Published:** 2017-06-22

**Authors:** Guillaume Morel, Joy Mootien, Philippe Guiot, Khaldoun Kuteifan

**Affiliations:** ^1^Service d'Hématologie, CHU de Strasbourg, 67000 Strasbourg, France; ^2^Service de Réanimation Médicale, GHRSMA, 68100 Mulhouse, France

## Abstract

TAFRO syndrome is a distinct idiopathic multicentric Castleman disease characterized by the association of thrombocytopenia, anasarca, fever, reticulin fibrosis, and organomegaly. We report the first case occurring in a Caucasian pregnant woman. At 34 weeks of gestation, our patient presented with all clinical and biological symptoms compatible with a TAFRO syndrome. Tough quick cesarean section was performed as symptoms got worse with onset of multiorgan failure requiring mechanical ventilation for acute respiratory distress, continuous renal replacement, and vasopressors. Nine days after ICU admission, steroid boluses were started and allowed spectacular clinical and biological improvement. As systemic inflammatory manifestations are important, TAFRO syndrome can be mistaken with severe autoimmune diseases, systemic infections, hematological malignancies, or hemophagocytic lymphohistiocytosis.

## 1. Background

Castleman disease is a nontumoral lymphoproliferative disorder initially described by Castleman and Towne in 1954 [1] usually characterized as either unicentric (UCD) or multicentric Castleman disease (MCD). UCD is an isolated lymph adenopathy without symptoms except those due to the tumoral mass while MCD is defined by the presence of diffuse lymph adenopathy and hepatosplenomegaly associated with systemic inflammatory manifestations: fever, asthenia, and weight loss. Laboratory abnormalities commonly include elevated inflammatory markers, microcytic anemia, hypergammaglobulinemia, and hypoalbuminemia [2]. Coombs test can also be positive. MCD is highly associated with HIV infection and these patients are also coinfected by HHV8 [3]. There is also a cohort of MCD patients without HIV and HHV8 coinfection defined as idiopathic multicentric Castleman disease (iMCD) [4]. TAFRO syndrome is a distinct subtype of iMCD characterized by the association of thrombocytopenia, anasarca, fever, reticulin fibrosis, and organomegaly [5]. The first description of TAFRO syndrome was published in 2010 by Takai et al. [6] who reported the case of three patients presenting these symptoms with histological findings compatible with iMCD. Some case reports or case series followed this first description. We report hereby the first case of TAFRO syndrome occurring in a Caucasian pregnant woman.

## 2. Case Report

A Caucasian 24-year-old woman, gravida 1, para 1 at 34 weeks of gestation, presented to our obstetric emergency department. The patient had no medical history and the pregnancy period was going well. One week before admission to our hospital, she noticed progressive weight gain associated with bilateral lower leg oedema. She also reported dyspnea, chest tightness, anorexia, and asthenia. The blood pressure was normal. Clinical exam also revealed hyperreflexia, hepatosplenomegaly, and diffuse supracentimetric lymph adenopathy. Laboratory tests were remarkable for moderate thrombocytopenia (138 × 10^9^/L) and hyponatremia (125 mmol/L) as well as an increase of C-reactive protein (194 mg/L) and uric acid (607 *µ*mol/L). Renal impairment was not observed though the patient was oliguric. Dipstick urine test did not reveal proteinuria.

**Figure 1:**
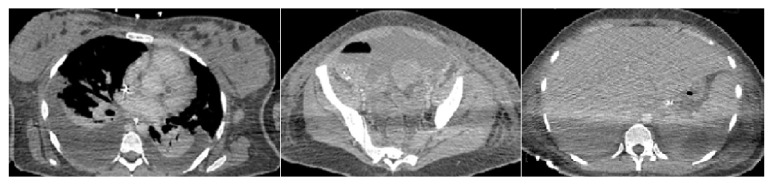
Computerized tomography scan showing bilateral pleural effusion and ascites. Presence of a right axillary adenopathy and hepatosplenomegaly.

Finally, three days after her admission a cesarean section was performed due to the bad tolerance. She gave birth to a girl weighting 2,650 grams with Apgar scores of 3 and 9. The surgery was smooth but the presence of ascites was noticed. The next days, chest pain and hyperreflexia disappeared but edema got worse with a total weight gain of 10 kg. Respiratory distress came out and the patient was oliguric despite diuretics as well as fever and thrombocytopenia got deeper to 50 × 10^9^/L associated with epistaxis. Computerized tomography scan showed diffuse superficial and deep lymphadenopathy, pleural effusions, ascites, and massive hepatosplenomegaly (Figure 1).

Two days later the patient was transferred to our intensive care unit, requiring mechanical ventilation for acute respiratory distress and continuous renal replacement for acute renal failure. She also became hypotensive and needed pressor support. Hemodynamic monitoring revealed hyperdynamic and vasoplegic profile. Myocardial function was preserved at transthoracic echocardiography.  Table 1 shows the evolution of biological parameters during ICU hospitalization.

**Figure 2:**
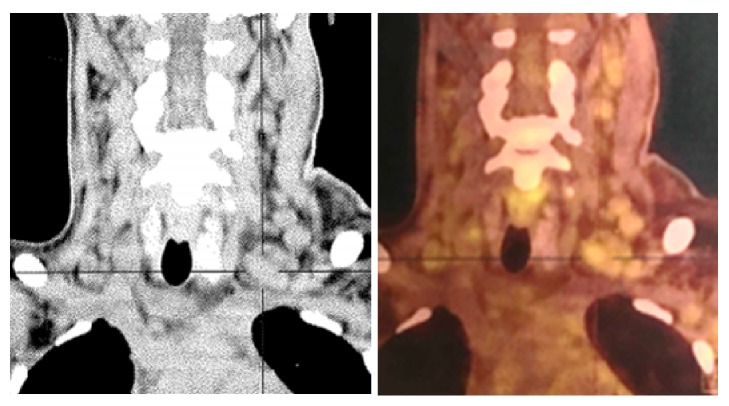
Positron emission tomography using [18F] fluorodeoxyglucose showing diffuse infracentimetric supra and infradiaphragmatic adenopathy with low metabolic activity.

First-line large spectrum antibiotics (Piperacillin-Tazobactam and Ciprofloxacin) were started but fever and inflammatory syndrome kept going. A second line antibiotic was then introduced (Meropenem, Vancomycin, and Amikacin) together with Caspofungin two days later. All blood, protected samples of lower respiratory tract secretions and urinary cultures were sterile. Serological tests for HIV, Hepatitis B and C virus, CMV (for both IgM and IgG), EBV (for both IgM and IgG), HTLV1, and Parvovirus were negative. PCR blood test did not reveal viral replication for CMV and HHV8. Exhaustive immune tests were also not contributive, and the serum complement was normal. Neither monoclonal nor polyclonal elevation of gamma globulin was found. Only serum level of IL-6 was increased (842 pg/mL). Bone marrow biopsy revealed a hypercellular marrow with megakaryocytic hyperplasia associated with the presence of reticulin fibrosis. Tumoral burden was assessed by positron emission tomography using [18F] fluorodeoxyglucose and showed diffuse supra- and infradiaphragmatic adenopathy. All tumoral lymph nodes were smaller than two centimeters and the metabolic activity was low (Figure 2).

The diagnosis of TAFRO-like syndrome was proposed. Nine days after ICU admission, steroid boluses of 1 g of methylprednisolone per day during three days were started followed by 1,5 mg/kg/day.

Spectacular clinical and biological improvement followed steroid introduction. Fever disappeared and blood pressure became normal allowing the weaning of pressor support. We also noticed a decline of pleural effusions and ascites which conduced to respiratory improvement and we preceded extubation eleven days later. Dialysis was stopped at day 30 as the renal function improved. Tumoral infiltration also declined. ICU acquired neuromyopathy was observed but got better with intensive kinesitherapy. Finally thirty-eight days after ICU admission the patient was transferred to internal medicine unit and rehabilitation started.

## 3. Discussion

Diagnostic criteria of TAFRO syndrome [7] include presence of histological criteria (compatible with pathological findings of lymph node as TAFRO-iMCD and negative LANA-1 for HHV8), three of five major criteria (thrombocytopenia, anasarca, fever, reticulin fibrosis, and organomegaly), and one of two minor criteria (hyper/normoplasia of megakaryocytes in bone marrow or high levels of serum ALP without markedly elevated serum transaminase) which are needed to meet the diagnosis of TAFRO syndrome. Our patient met all the major and minor criteria but unfortunately we could not have a histological analysis of a lymph adenopathy. However we had considered this diagnostic and started treatment by steroids. Moreover we quickly observed a significant improvement of hemodynamic, respiratory, and hematological and renal parameters which strengthened our diagnosis.

As systemic inflammatory manifestations are important, TAFRO syndrome can be mistaken with severe autoimmune diseases [8], systemic infections, hematological malignancies, or hemophagocytic lymphohistiocytosis. These syndromes were proposed but none of them were absolutely compatible with the diagnostic. There was no evidence for underlying autoimmune disease which could explain systemic inflammation or renal failure. C-reactive protein was high but all microbiological cultures serology and PCR were negative and ruled out a possible systemic infection. On the other hand, a tumoral syndrome was present but bone marrow biopsy did not find tumoral infiltration and PET scanner revealed small lymph nodes with low metabolic activity and consequently not compatible with an aggressive lymphoproliferative disorder. Furthermore *H*-score [9] was not increased, particularly due to the low ferritin level, and excluded the probability of hemophagocytic lymphohistiocytosis. Finally only TAFRO-like syndrome was the compatible diagnostic.

First-line steroid is the usual treatment for TAFRO syndrome [10] and can bring about sustained remissions. In case of relapse or refractory disease, several immunosuppressive drugs have been proposed and also associated with successful disease control. For example Cyclosporine A [11] may be an alternative therapy for refractory TAFRO syndrome. Anti-IL-6 receptor monoclonal antibodies such as Tocilizumab have demonstrated effectiveness [12] leading to durable remission. Cytotoxic chemotherapies based on lymphoma protocols showed responses in TAFRO syndrome [13] or iMCD patients but is associated with chemotherapy-linked side effects and relapse is common. Rituximab, which is also used in iMCD, may be effective for disease control in TAFRO syndrome [14].

## Figures and Tables

**Table 1:** Evolution of biological tests in ICU.

	Day 0	Day 4	Day 7	Day 9	Day 12	Day 16	Day 20	Day 30
	Admission in ICU	Pressor support	Dialysis	Steroids		Stop pressor support	Stop ventilation	Stop dialysis
Leucocyte count (G/L)	17,3	27,9	24,1	36,4	38,1	23,5	27,9	19,8
Hemoglobin (g/dL)	9,2	6,9	8,6	8,1	7,6	7,9	8,2	7,8
Platelets (G/L)	54	30	26	22	30	59	183	116
Serum creatinine (*µ*mol/L)	75	137	151	153	99	86	73	60
Fibrinogen (mg/dL)	930	780	790	530	520	640	610	410
C-reactive protein (mg/dL)	34,3	28,9	31,4	29,5	21,4			2,9
Serum ferritin (ng/mL)	526	939		1053				261
Triglycerides (mg/dL)	240							430
Albumin (g/dL)	1,6	1,8	2	2,4	2,5			2,9
Aspartate amino-transferase (IU/L)		7	6	9	43	134	46	35
Alanine amino-transferase (IU/L)		21	32	52	131	155	81	18
Alkaline phosphatase (IU/L)		479	355	176	204	655	949	261
Lactate dehydrogenase (IU/L)		240	443	497	505			161
Blood lactate (mmol/L)	1,25	0,6	2,7	2,7	2,2	2,1	1,4	3

*Reference Ranges*. Leucocyte count (4,0–10 G/L), hemoglobin (11–16 g/dL), platelets (150–450 G/L), serum creatinine (45–90 *µ*mol/L), fibrinogen (200–400 mg/dL), C-reactive protein (0–0,4 mg/dL), serum ferritin (12–180 ng/mL), triglycerides (43–148 mg/dL), albumin (3,2–5,5 g/dL), aspartate amino-transferase (0–40 (IU/L)), alanine amino-transferase (0–40 IU/L), alkaline phosphatase (115–359 IU/L), lactate dehydrogenase (85–250 IU/L), and blood lactate (0,6–2,1 mmol/L).
